# Editorial: Natriuretic Hormones

**DOI:** 10.3389/fendo.2015.00108

**Published:** 2015-07-16

**Authors:** Harvey Craig Gonick, Vardaman M. Buckalew

**Affiliations:** ^1^Division of Nephrology, Department of Medicine, David Geffen School of Medicine, University of California Los Angeles, Los Angeles, CA, USA; ^2^Section of Nephrology, Department of Medicine, Wake Forest School of Medicine, Winston Salem, NC, USA

**Keywords:** natriuretic hormones, atrial natriuretic peptides, cardiotonic steroids, hypertension, endogenous ouabain, bufodienolides

The link between renal sodium excretion and body fluid volumes has been a topic of interest since the early days of renal physiology (1). Glomerular filtration rate (GFR) and aldosterone were the first two controllers of renal sodium excretion to be recognized. A “third factor” was discovered when de Wardener and colleagues showed that volume expansion natriuresis still occurred in dogs given supramaximal doses of mineralocorticoids and without an increase in GFR (2). de Wardener et al. suggested that the natriuresis was due to a “natriuretic hormone” (NH), launching a new field of investigation. NH was thus defined as a compound that circulates in blood, the level of which is regulated appropriately by changes in sodium and water balance.

The nine chapters that follow highlight several areas in which the NH field has subsequently developed. First, de Wardener’s original experiments were refined to control potential factors other than GFR and aldosterone. These studies, reviewed by Lichardus (3), confirmed the original observation, and also led to the discovery of the so-called “physical factors” that affect sodium excretion (4). Second, attempts to purify NH from various tissues and body fluids, as summarized in Table 1 by Hamlyn (5), led to the discovery of two important families of factors, atrial natriuretic peptides (ANP), and endogenous cardiotonic steroids (CTS).

The discovery of ANP led rapidly to its characterization as a family of peptides with three major components: ANP derived from cardiac tissue, BNP from brain, and CNP from endothelium (6). This complex system of natriuretic vasodilators, discussed in the chapters by Hamlyn (5), and Hodes and Lichstein (6), continues to be of interest as an NH, a neurotransmitter (6), and a factor with many other functions (7).

In sharp contrast, the identification of endogenous CTS as circulating Na, K ATPase inhibitors has been the source of considerable controversy (8). Two structural classes of CTS have been identified: authentic ouabain, and the bufodienolides, originally identified in toads (9). As discussed by Hamlyn, a bufodienolide is the more likely candidate NH, whereas ouabain in physiologic concentration appears to be antinatriuretic (5). CTS cause vasoconstriction by a mechanism proposed by Blaustein et al. (10) and endogenous CTS have been implicated in the pathophysiology of hypertension (9). Despite overwhelming evidence to the contrary, however, difficulty identifying ouabain in biological fluids continues to be reported (11, 12).

At least three other natriuretic compounds related to control of sodium balance have been identified. As discussed by Gonick (13), a natriuretic protein associated with various forms of human hypertension consists of a 408 Da compound and a 12 kDa carrier protein. The identity of these compounds has not been completed (see below). Two xanthurenic acid derivatives (MW 368 and 284), discussed by Bricker et al. (14), are potential regulators of sodium balance in chronic renal failure. Dietary sodium releases two natriuretic factors from the gastrointestinal tract identified as guanylin and uroguanylin (MW 10.3 and 1.7 kDa), discussed in the paper by Hodes and Lichstein (6).

Two other types of natriuretic compounds that inhibit Na, K ATPase, but are not natriuretic hormones as defined above, are two vanadium diascorbates (MW approximately 400), and two spherical oligo silicic acids (SOSA) (MW 408). These interesting compounds of uncertain significance are described in papers by their discoverers Kramer (15) and Kerek and Voicu (16), respectively. The apparent identical molecular weight of SOSA and the compound isolated by Gonick is possibly a coincidence. Alternatively, it may be related to the trace amounts of silicon in human plasma (17) and have significance beyond that implied by the serendipitous discovery of SOSA.

A third area is studies of the physiological mechanism(s) by which these factors might function as natriuretic hormones. ANP release is controlled by stretch of the cardiac atria (18), and in that regard is a classic, volume controlled, NH system as originally conceived (19). Although evidence indicates that CTS are released in response to increased sodium intake (5), no causal mechanism connecting sodium intake and CTS release has been demonstrated. As noted by Hamlyn (5), cerebrospinal fluid sodium concentration controls the release of an unidentified natriuretic factor from brain, causing “CNS natriuresis.” The possible role of CTS or some other NH in this phenomenon should be explored.

A fourth area of investigation is studies of collateral, pathophysiological, effects of the various factors. Both ANP and CTS are vasoactive and have been implicated in the pathophysiology of hypertension (5, 6, 9). Since both appear to be neurotransmitters or neuromodulators (6), their role in hypertension may have both central and peripheral components. Pathological effects of excess CTS in the CNS may also include an etiologic role in mood disorders, including depression and bipolar disorder (6).

Finally, the signaling effects of CTS on Na, K ATPase not dependent on ion pumping, mediated by activation of the tyrosine kinase Src and other signaling molecules, is discussed by Xie et al. (20). This pathway is involved in the natriuretic effect of bufodienolides (21), and is implicated in pathophysiological processes, such as oxidative stress, and organ fibrosis (20).

de Wardener’s original hypothesis has led to the discovery of a rich array of factors with multiple biologic activities. ANP is the only classical NH described so far. Future work, including the development of methods for antagonizing these factors, should increase our understanding of the regulation of renal sodium excretion and blood pressure and offer novel treatments for several clinical disorders.

## Conflict of Interest Statement

The authors declare that the research was conducted in the absence of any commercial or financial relationships that could be construed as a potential conflict of interest.
